# Establishment of a novel glycolysis-immune-related diagnosis gene signature for endometriosis by machine learning

**DOI:** 10.1007/s10815-023-02769-0

**Published:** 2023-03-17

**Authors:** Qizhen Chen, Yufan Jiao, Zhe Yin, Xiayan Fu, Shana Guo, Yuhua Zhou, Yanqiu Wang

**Affiliations:** 1 Reproductive Medical Center, Department of Gynecology and Obstetrics, Tongji Hospital, Tongji University School of Medicine, Shanghai, China; 2 Department of Ultrasonography, Tongji Hospital, Tongji University School of Medicine, Shanghai, China

**Keywords:** Endometriosis, Glycolysis, Immune infiltration, Machine learning, Diagnosis

## Abstract

**Purpose:**

The objective of this study was to investigate the key glycolysis-related genes linked to immune cell infiltration in endometriosis and to develop a new endometriosis (EMS) predictive model.

**Methods:**

A training set and a test set were created from the Gene Expression Omnibus (GEO) public database. We identified five glycolysis-related genes using least absolute shrinkage and selection operator (LASSO) regression and the random forest method. Then, we developed and tested a prediction model for EMS diagnosis. The CIBERSORT method was used to compare the infiltration of 22 different immune cells. We examined the relationship between key glycolysis-related genes and immune factors in the eutopic endometrium of women with endometriosis. In addition, Gene Ontology (GO)-based semantic similarity and logistic regression model analyses were used to investigate core genes. Reverse real-time quantitative PCR (RT-qPCR) of 5 target genes was analysed.

**Results:**

The five glycolysis-related hub genes (CHPF, CITED2, GPC3, PDK3, ADH6) were used to establish a predictive model for EMS. In the training and test sets, the area under the curve (AUC) of the receiver operating characteristic curve (ROC) prediction model was 0.777, 0.824, and 0.774. Additionally, there was a remarkable difference in the immune environment between the EMS and control groups. Eventually, the five target genes were verified by RT-qPCR.

**Conclusion:**

The glycolysis-immune-based predictive model was established to forecast EMS patients’ diagnosis, and a detailed comprehension of the interactions between endometriosis, glycolysis, and the immune system may be vital for the recognition of potential novel therapeutic approaches and targets for EMS patients.

**Supplementary Information:**

The online version contains supplementary material available at 10.1007/s10815-023-02769-0.

## Background

Endometriosis is a chronic inflammatory illness in which endometrial tissue outside the uterus causes pelvic pain and infertility [1]. Endometriosis is a condition that affects 5–10% of reproductive-aged women worldwide. Despite its prevalence, the rate of misdiagnosis is high. Most women have difficulties expressing their symptoms or believing that their symptoms are being normalized in an unsuitable way [2]. Furthermore, the current requirement for surgical diagnosis—typically via diagnostic laparoscopy—creates a barrier to early detection and treatment [2]. Therefore, an urgent need exists to create a reliable prognostic prediction model for patients in the early phase through a noninvasive method.

Although there are numerous theories to consider explaining the causes of endometriosis, the explanation of retrograde menstruation proposed by Sampson is the most widely accepted [3]. It claims that fragments of monthly endometrial tissue, including viable endometrial glands and stroma, are retrogradely expelled into the peritoneal cavity via the fallopian tubes, where they cling to and infect the underlying mesothelium [3]. Other factors, on the other hand, are required to promote endometrial stromal and glandular cell invasion and proliferation, including changes in the immune environment, reprogramming of glucose metabolism, and local complex hormone effects [4].

It has long been known that cells rely on glycolysis to generate energy. From the view of evolution, cells grow in an anaerobic environment and can tolerate anaerobic glycolysis, so glycolysis is considered to be the oldest ATP production pathway [5]. Recent studies have revealed the benefits and specific advantages of aerobic glycolysis. Although glycolysis produces less ATP than the tricarboxylic acid oxidative phosphorylation pathway, proliferative cells prefer glycolysis for several reasons [6]. First, glycolysis and the conversion of glucose to lactate are increased, resulting in faster and greater ATP generation [7, 8]. Glycolytic ATP generation could be 100 times faster than the oxidative phosphorylation of tricarboxylic acids [9]. Meanwhile, the intracellular need is met by the modest synthesis of ATP from glycolysis. In conclusion, glycolysis may confer a selective growth advantage to proliferative cells [10, 11].

Compared to epithelial cells and stromal cells in the normal endometrium, previous studies have indicated that both have higher proliferation, adhesion, and invasion abilities [12–14]. Elevated levels of glycolysis (the Warburg effect) can lead to lactate production and substance synthesis. Lactate accumulation promotes tumour cell migration, invasion, angiogenesis, and immune escape [15, 16].

Interestingly, all the above cancer-like processes are also involved in the survival and invasion of eutopic endometrial cells, thus contributing to the development of endometriosis [17].

A growing body of research suggests a link between glycolysis and immunological evasion [18]. Whilst EM has benign clinical and pathological symptoms, it has cancer-like features such as spread, invasion, and hyperplasia [19]. Cancers with a significant Warburg effect develop a tumour microenvironment (TME) deprived of glucose, limiting local immune surveillance via nutritional competition [20]. Meanwhile, immune cells can promote glycolysis in the same way that tumour cells can. Previous research has found that the immunological environment of the eutopic endometrium in women with endometriosis differs from that of the normal endometrium, but in endometriosis, the relationship between glycolysis and the immunological milieu is poorly understood [21].

In conclusion, we wanted to create a model of endometriosis linked to glycolysis and investigate its connection with the immune microenvironment. The Gene Expression Omnibus database was used to obtain gene chips. Least absolute shrinkage and selection operator (LASSO) and random forest (RF) were used to identify five prognostically related glycolytic genes. Next, the association between the eutopic endometrium immune environment and key genes was investigated, and a logistic regression model was built by ROC and verified by the test set. These findings could help researchers and clinicians better understand EMS.

## Materials and methods

### Gene expression data acquisition

**Fig. 1:**
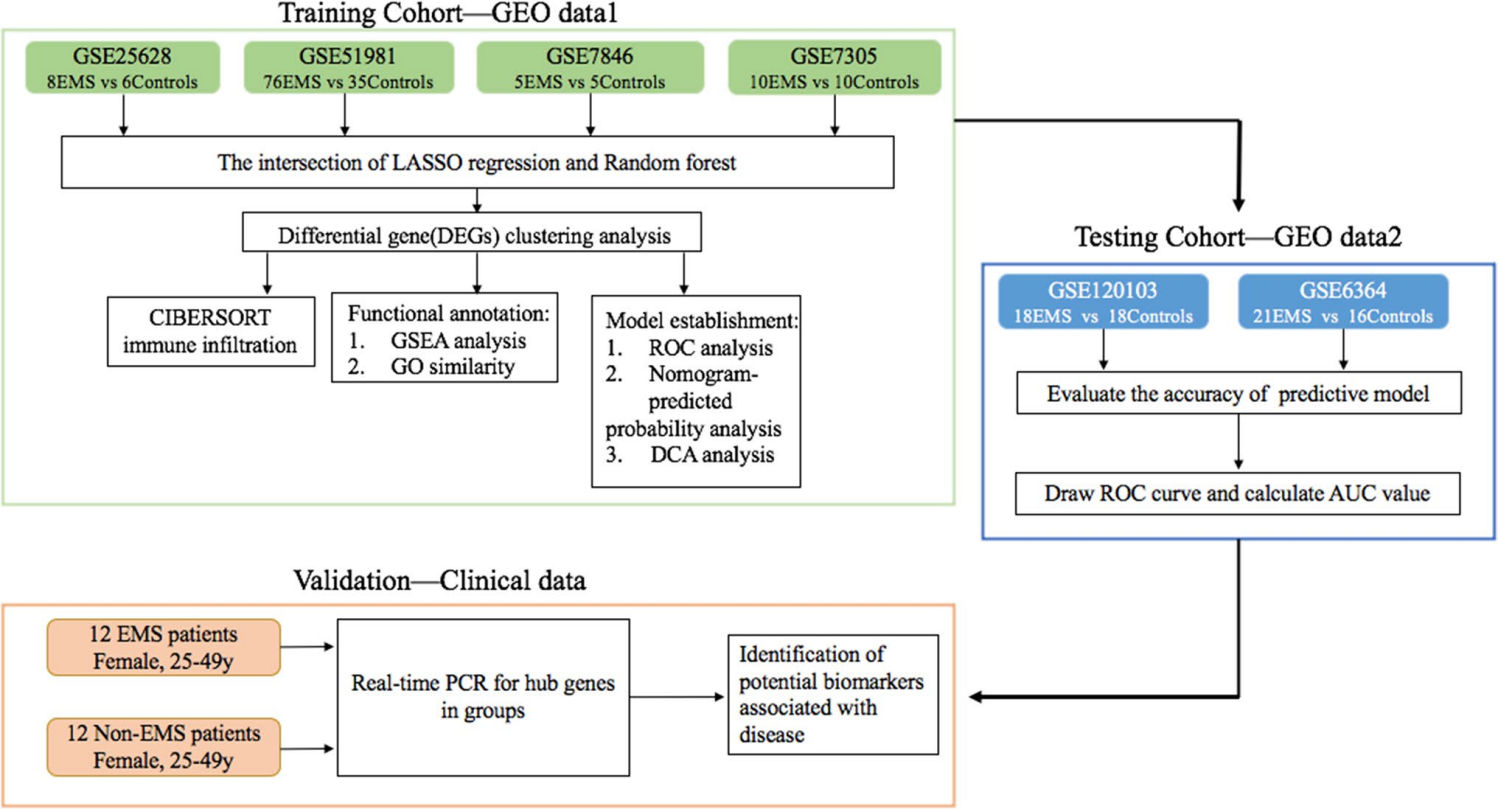
Overview of the study workflow

GEO was used to download RNA-sequence profiles and data with and without endometriosis. The following are the eligibility requirements: first, eutopic endometrium samples collected from endometriosis patients and healthy controls; second, samples having glandular and stromal components; and last, the females in the study were in the proliferative and early secretory phases of the menstrual cycle. GSE25628 (8 eutopic endometria and 6 healthy controls), GSE51981 (76 eutopic endometria and 35 healthy controls), GSE7846 (5 eutopic endometria and 5 healthy controls), and GSE7305 (10 eutopic endometria and 10 healthy controls) were included in the study as the training set. Four training datasets from GPL570 were analysed by Affymetrix Human Genome U133 Plus 2.0 Array. The test set comprised GSE120103 (18 eutopic endometria and 18 healthy controls) and GSE6364 (21 eutopic endometria and 16 healthy controls) to confirm our predictive model (Table 1). Two datasets from GPL6480 and GPL570 were used as the validation cohorts. GPL6480 was analysed by Agilent-014850 Whole Human Genome Microarray 4 × 44 K G4112F. For normalization, we utilized the “sva” utility in R to remove disparities across batches because our datasets came from diverse cohorts and array platforms. For further analysis, we obtained 15,926 common genes (Fig. 1).

**Table 1:** The RNA-sequence profiles used in this study

GEO accession	Platform	Experiment type	EM (*N*)	Normal (*N*)	Tissue	Year	Dataset
GSE25628	GPL571	mRNA array	8	6	Endometrium	2010	Training
GSE51981	GPL570	mRNA array	76	35	Endometrium	2013	Training
GSE7846	GPL570	mRNA array	5	5	Endometrium	2007	Training
GSE7305	GPL570	mRNA array	10	10	Endometrium	2007	Training
GSE120103	GPL6480	mRNA array	18	18	Endometrium	2019	Test
GSE6364	GPL570	mRNA array	21	16	Endometrium	2007	Test

### Glycolysis-related gene sets

The Molecular Signatures Database (MSigDB) is a library of annotated gene sets for Gene Set Enrichment Analysis (GSEA). Five gene sets relevant to glycolysis were retrieved, including BIOCARTA_GLYCOLYSIS_PATHWAY, BIOCARTA_FEEDER_PATHWAY, HALLMARK_GLYCOLYSIS, GO_GLYCOLYTIC_PROCESS, and REACTOME_GLYCOLYSIS. Using the “limma” program in R software, we found 262 glycolysis-related genes in 155 cases.

### Identification and validation of predictive gene signature

The glycolysis-related diagnostic indicators of EMS were classified using LASSO logistic regression and random forest. The “glmnet” program was used to conduct the LASSO analysis, with the response type set to binomial and the alpha set to 1. Random forest is a technique that uses recursive partitioning to generate a binary tree. The random forest method was given a number of trees of 500. Then, we chose the top 20 genes from the RF analysis to interact with the LASSO results and used Venn diagram to visualize the intersection of gene lists.

### Construction of the EMS diagnostic model

Machine algorithm logistic regression (LR), random forest (RF), and lasso regression (LASSO) were used to construct the diagnostic model of EMS based on the glycolysis-related diagnostic markers. The following is how a model was created:

$$Risk\;score=\exp\_gene\_1\times coef\_gene\_1+expr\_gene\_2\times coef\_gene\_2+\dots+expr\_gene\_n\times coef\_gene\_n$$

The model’s effectiveness and accuracy were assessed using ROC curves and AUC values. The nomogram’s accuracy was assessed using calibration plots. The best predicted value was indicated by the 45° line. The more perfect the result, the closer the curve was. The clinical utility of this model was examined using decision curve analysis (DCA).

### Evaluation of immune cell subtype distribution

The CIBERSORT algorithm was used to infer the relative proportion of 22 different types of immuno-infiltrating cells from RNA-seq data of women with and without EMS. Gene expression and immune-cell content were subjected to Spearman correlation analysis. A statistically significant value was defined as *p* < 0.05.

### Gene Set Enrichment Analysis

Based on the expression of the five hub genes, EMS patients were categorized into two groups: high and low. GSEA of the two groups was accomplished through the use of signal pathway differences. The background gene set data were obtained from the Molecular Signature Database. Maximum (500) and minimum gene sets were used to select the gene set. Enriched gene sets were found after 1000 permutations with a *p* < 0.05 cut-off. The significantly enriched gene sets were then sorted in order of their significance. GSEA was used to investigate the relationships between various expression groups and biological processes.

### Semantic similarity GO annotations

We used the GO Semsim software package of Wang’s method to explore the functional similarity between proteins [22]. In terms of molecular function (MF), biological processes (BP), and cellular component (CC), we calculated the geometric mean of GO semantic similarity. To measure functional similarity, the geometric average of semantic similarity was utilized.

### Coexpression analysis of the hub genes

The “corrplot” and “circlize” tools in R software were used to perform correlation analysis. The corrplot tool in R was used to plot the Pearson correlation of hub gene expression (version 1.64). The circlize package was used to generate circos plots. The colours “red” and “green” represent correlation coefficients. A positive correlation is indicated by the red colour, whereas a negative correlation is indicated by the green colour. The stronger the relationship, the darker the colour and thicker the cord.

### Human subjects and sample collection

Eutopic endometrial tissues (*n* = 12) were obtained from women with ovarian endometriotic cysts undergoing laparoscopic surgery at the Tongji Hospital of Tongji University from August to December 2021, and control endometrial tissues (*n* = 12) were obtained from patients without endometriosis who underwent hysterectomy for uterine leiomyoma. All of the tissue samples were confirmed through histological examination. Exclusion criteria included all women unable to consent, those under the age of 18, currently pregnant, malignancy of any kind, acute inflammatory disease or infection, and systemic autoimmune disease [23]. All of the patients had normal ovulation with regular menstrual cycles, and none of the patients had received any ovulation-promoting drugs in the 3 months before enrolment. The ethics committee of Tongji Hospital of Tongji University approved this study, and informed consent was obtained from all participants.

### RT-qPCR validation of the hub genes

RNA was extracted using Vazyme RNA Isolater Total RNA Extraction Reagent according to the manufacturer’s instructions. First-strand cDNA synthesis was performed using HiScript II Q RT SuperMix for qPCR (Vazyme). qPCR was carried out using the Taq Pro Universal SYBR qPCR Master Mix. GAPDH was used as an internal reference. The relative expression of target genes was calculated using the 2^−ΔΔCt^ method. A *p* value < 0.05 was considered significant. Primer sequences are summarized in Table 2. All PCRs were conducted in triplicate.

**Table 2:** Primer information

Target name		Primer
GAPDH	F	AATGGGCAGCCGTTAGGAAA
GAPDH	R	GCGCCCAATACGACCAAATC
CITED2	F	GCGAAGCTGGGGAATAACAAC
CITED2	R	TCTGCCATTTCCAGTCTTCAGG
CHPF	F	CTTCCCCTCATCTTAGGGCTG
CHPF	R	CATCACTTTGGTCTAGCCGAG
ADH6	F	GTGTGGTTGTTGGGGTGTTG
ADH6	R	CTGCTCTTCCAGCCTCCAAA
GPC3	F	CCTTTGAAATTGTTGTTCGCCA
GPC3	R	CCTGGGTTCATTAGCTGGGTA
PDK3	F	CGCTCTCCATCAAACAATTCCT
PDK3	R	CCACTGAAGGGCGGTTAAGTA

### Statistical analysis

For statistical analyses and visualization of results, R software (version 4.1.2) was utilized. A *p value* of less than 0.05 was judged statistically significant. Significant correlation coefficients were defined as those with an absolute value greater than 0.2 and a *p value* less than 0.05. To create the predictive model, the logistic regression technique was employed.

## Results

### Identification of five glycolysis-related hub genes

**Fig. 2:**
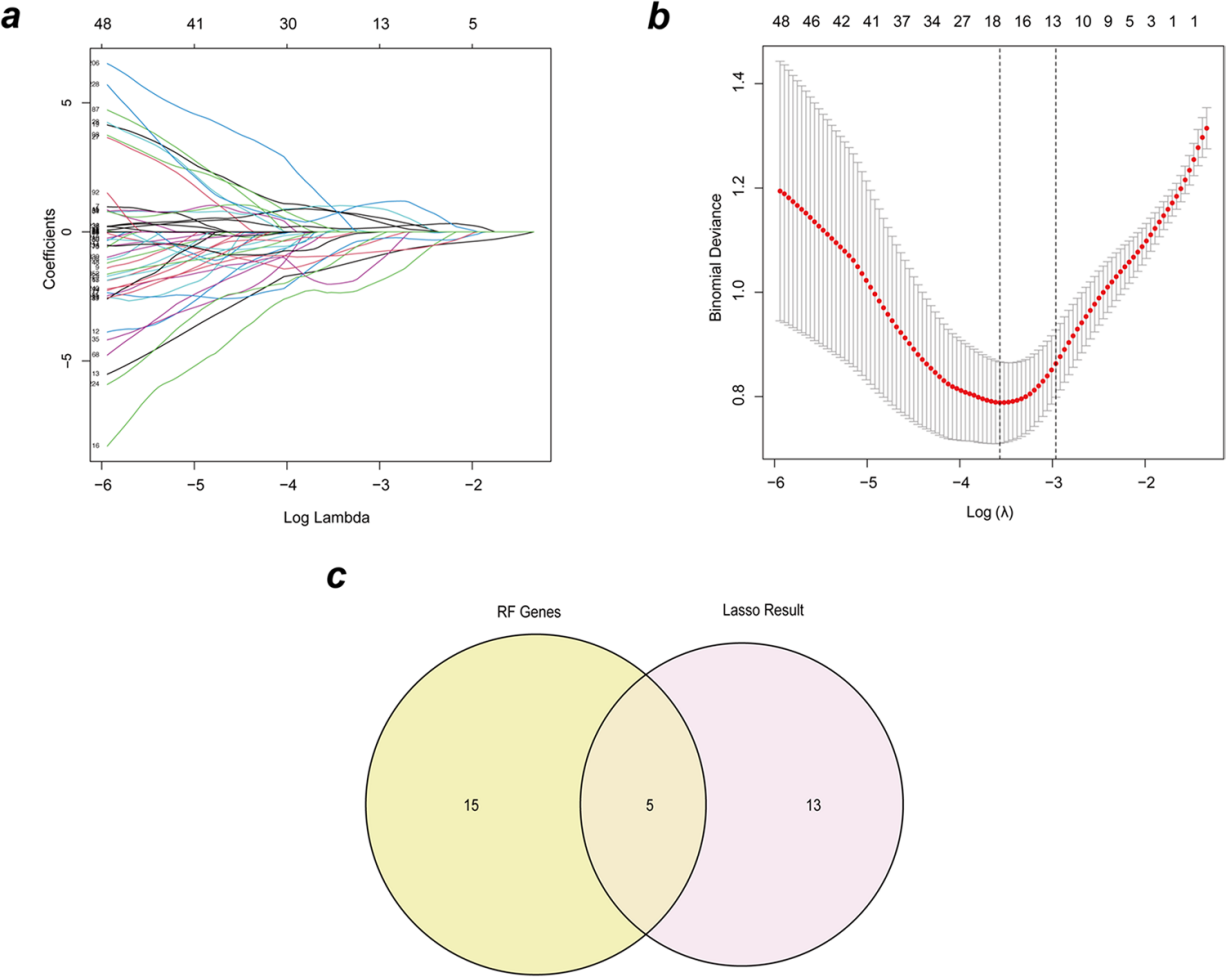
Selection of diagnostic biomarkers and identification of hub genes. **a** LASSO coefficient profiles of the 18 differentially expressed genes. **b** The misclassification error in the jackknife rates analysis. **c** Venn diagram of genes extracted from LASSO and RF methods

The training set was obtained from the NCBI GEO public database. There were 155 patients in total (EMS group, 99; control group, 56). The expression profiles of 262 glycolysis-related genes were derived using the differential expression profile. We used LASSO regression to perform feature screening to explore glycolysis-related biomarkers in EMS. LASSO regression revealed that 18 genes were signature genes. The 262 glycolysis-related DEGs were then fed into the random forest classifier. The variable relevance of the output results was quantified in terms of decreasing accuracy and decreasing mean square error during the construction of the random forest model. The top 20 DEGs, ranked in order of relevance, were then chosen as candidate genes for further investigation. The intersection of random forest genes and LASSO regression genes resulted in 5 DEGs (Fig. 2).

### Establishment and validation of the diagnostic model based on five glycolysis-related hub genes

We created a predictive model using the five genes below: chondroitin polymerizing factor (CHPF), Cbp/p300 interacting transactivator with Glu/Asp-rich carboxy-terminal domain 2 (CITED2), glypican 3 (GPC3), alcohol dehydrogenase 6 (ADH6), and pyruvate dehydrogenase kinase 3 (PDK3). The following risk model was created using coefficients for the five hub genes:

$$Risk\;score=\left(2.751\times CHPF\right)+\left(3.880\times PDK3\right)+\left(0.631\times CITED2\right)+\left(0.502\times GPC3\right)-(3.075\times ADH6)$$

**Fig. 3:**
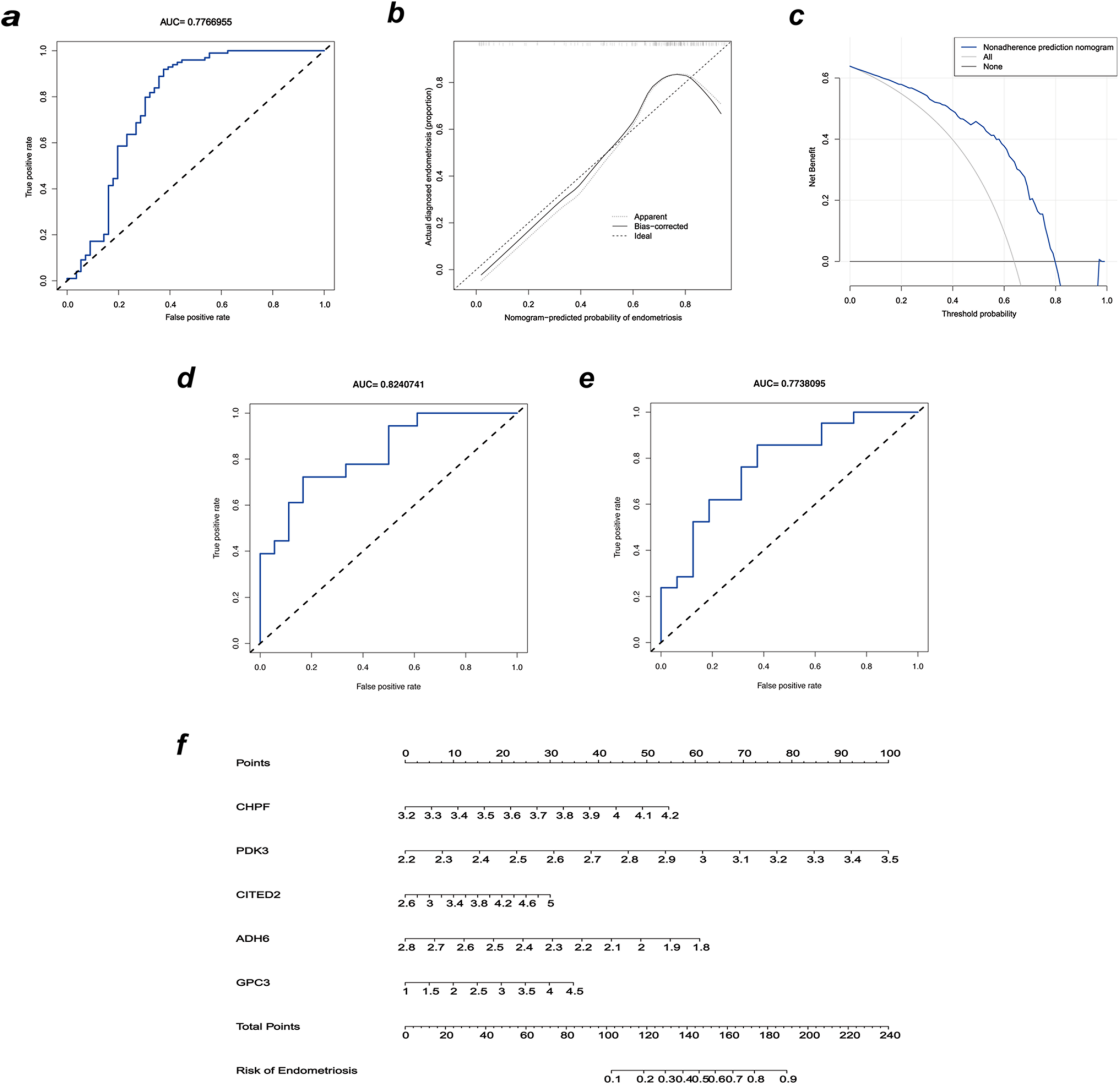
Establishment and verification of the glycolysis-immune-related diagnostic model for EMS. **a** ROC analysis of the glycolysis-immune-related diagnostic model using the training group. **b** Nomogram-predicted probability of EMS in the training group. **c** Decision curve analysis of the model in the training group. **d** and **e** ROC analysis of the glycolysis-immune-related diagnostic model using the test group (GSE120103 and GSE6364, respectively). **f** Nomogram for diagnosis of EMS

The performance of this model was examined using the area under the receiver operating characteristic (ROC) curve. In the training set, the area under the ROC curve (AUC) for this model was 0.777 (Fig. 3a), and the AUCs of the model in the test set were 0.824 and 0.774 (Fig. 3d, e). The calibration curve revealed that the model matched well with the actual and predicted probability of an EMS occurrence (Fig. 3b). The nomogram’s C-index for predicting the presence of EMS was 0.777 [95% confidence interval (CI): 0.727–0.827]. Furthermore, decision curve analysis (DCA) revealed that the anticipated and observed values were nearly identical (Fig. 3c). CHPF, CITED2, GPC3, ADH6, and PDK3 were used to create a diagnostic prediction model for EMS using a multivariable logistic regression model and are shown as a nomogram (Fig. 3f). The above results indicate the importance and independence of the risk score as a diagnostic model of EMS.

### The landscape of immune infiltration

**Fig. 4:**
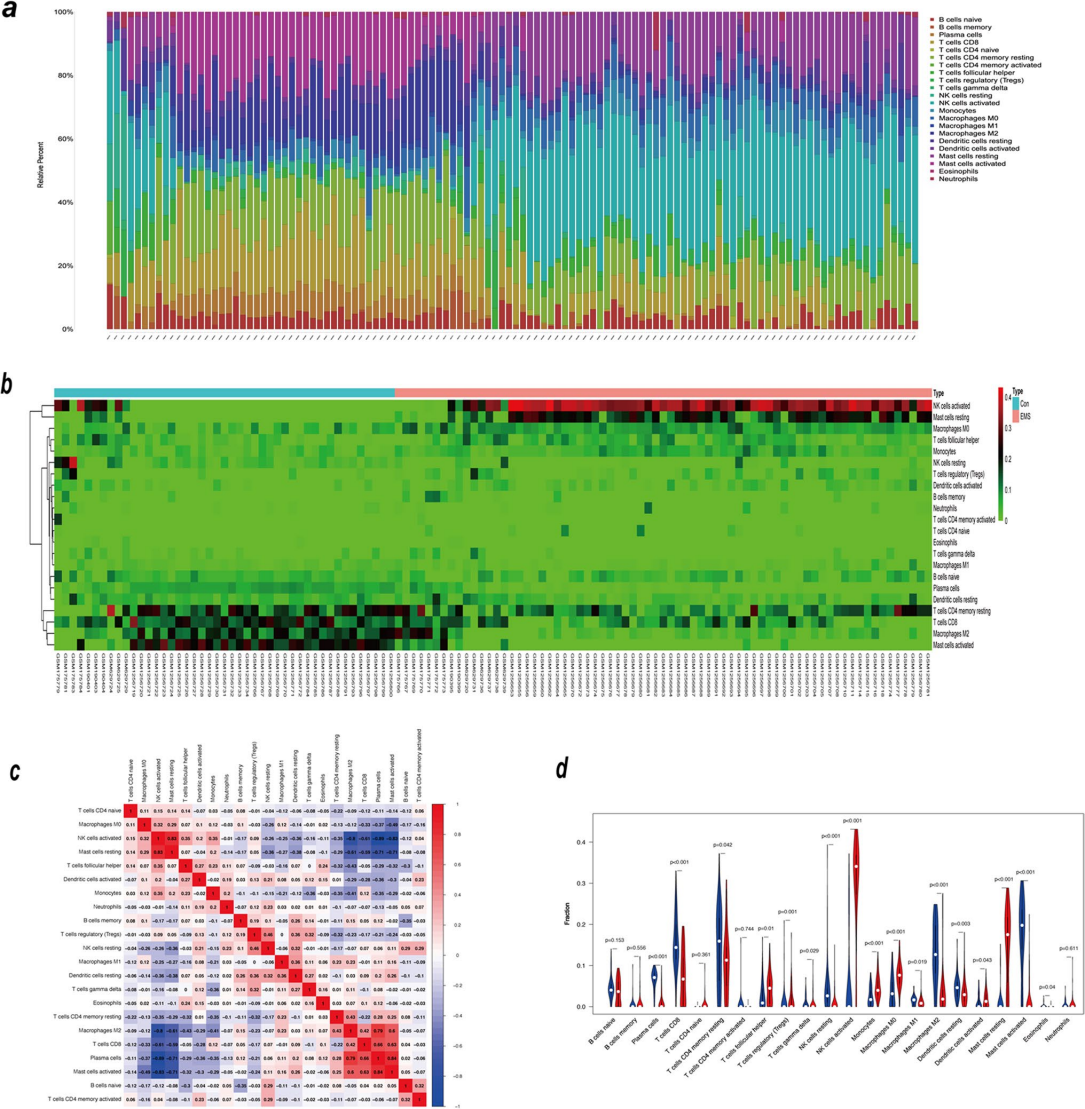
The landscape of immune infiltration between EMS and normal controls. **a** The box-plot diagram indicating the abundance ratio of immune cells in 116 samples. **b** The heatmap indicating the abundance ratio of immune cells in the EMS (*n* = 71) and control groups (*n* = 45). **c** The cor-heatmap shows the relationship between the abundance ratios of 22 immune cells. **d** The difference in immune infiltration between EMS (red) and normal (blue) controls (*p* values < 0.05 indicate statistical significance)

We employed the CIBERSORT algorithm to explore the difference between eutopic endometrium in endometriosis patients and healthy controls after revealing the landscape of infiltration of 22 immune cell subpopulations. The abundance ratios of 22 immune cells in the 155 samples are presented in Fig. 4a. The percentage of immune cells in each sample is shown in Fig. 4b. Figure 4c depicts the interaction of innate immune cells. Compared with the control endometrium, eutopic endometrium from women with endometriosis contained a greater number of follicular helper T cells, T cell regulators (Tregs), M0 macrophages, activated NK cells, monocytes, activated dendritic cells, and resting mast cells. However, this eutopic endometrium contained lower numbers of plasma cells, CD8 T cells, CD4 memory resting T cells, resting NK cells, M1 macrophages, M2 macrophages, resting dendritic cells, activated mast cells, gamma delta T cells, and eosinophils (Fig. 4d).

### Analysis of core genes and immune infiltration

**Fig. 5:**
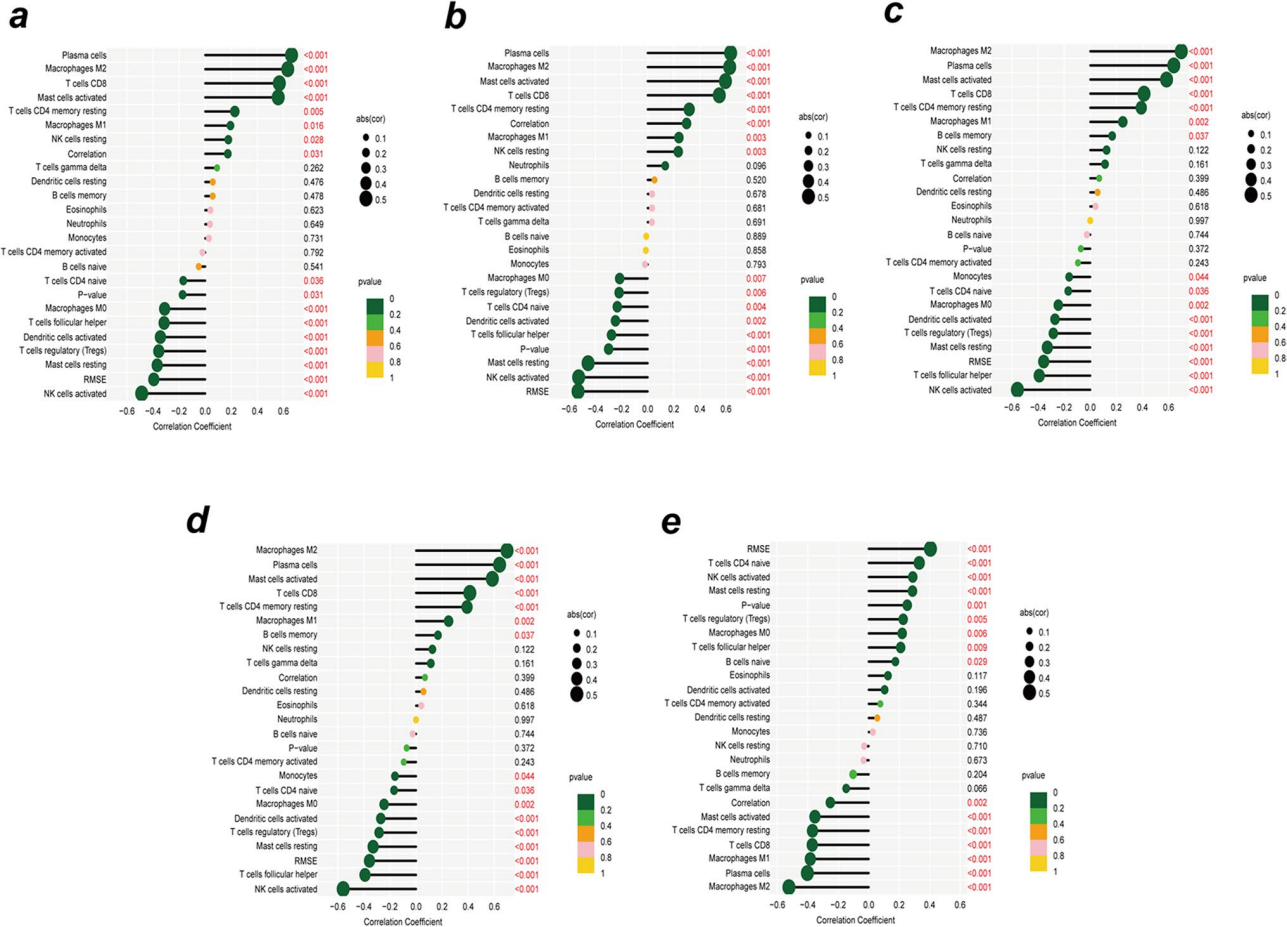
The association between the hub genes and the infiltration level: **a** CHPF, **b** CITED2, **c** GPC3, **d** PDK3, and **e** ADH6

When we further examined the interaction between hub genes and immune cells, the expression of risk hub genes (CHPF, CITED2, GPC3, PDK3) was shown to be positively connected with plasma cells, M2 macrophages, CD8 T cells, activated mast cells, and resting memory CD4 T cells. The protective hub gene (ADH6) had a positive correlation with naïve CD4 T cells, activated NK cells, resting mast cells, regulatory T cells (Tregs), and M0 macrophages (Fig. 5a–e).

### Analysis of core genes and immune factors

**Fig. 6:**
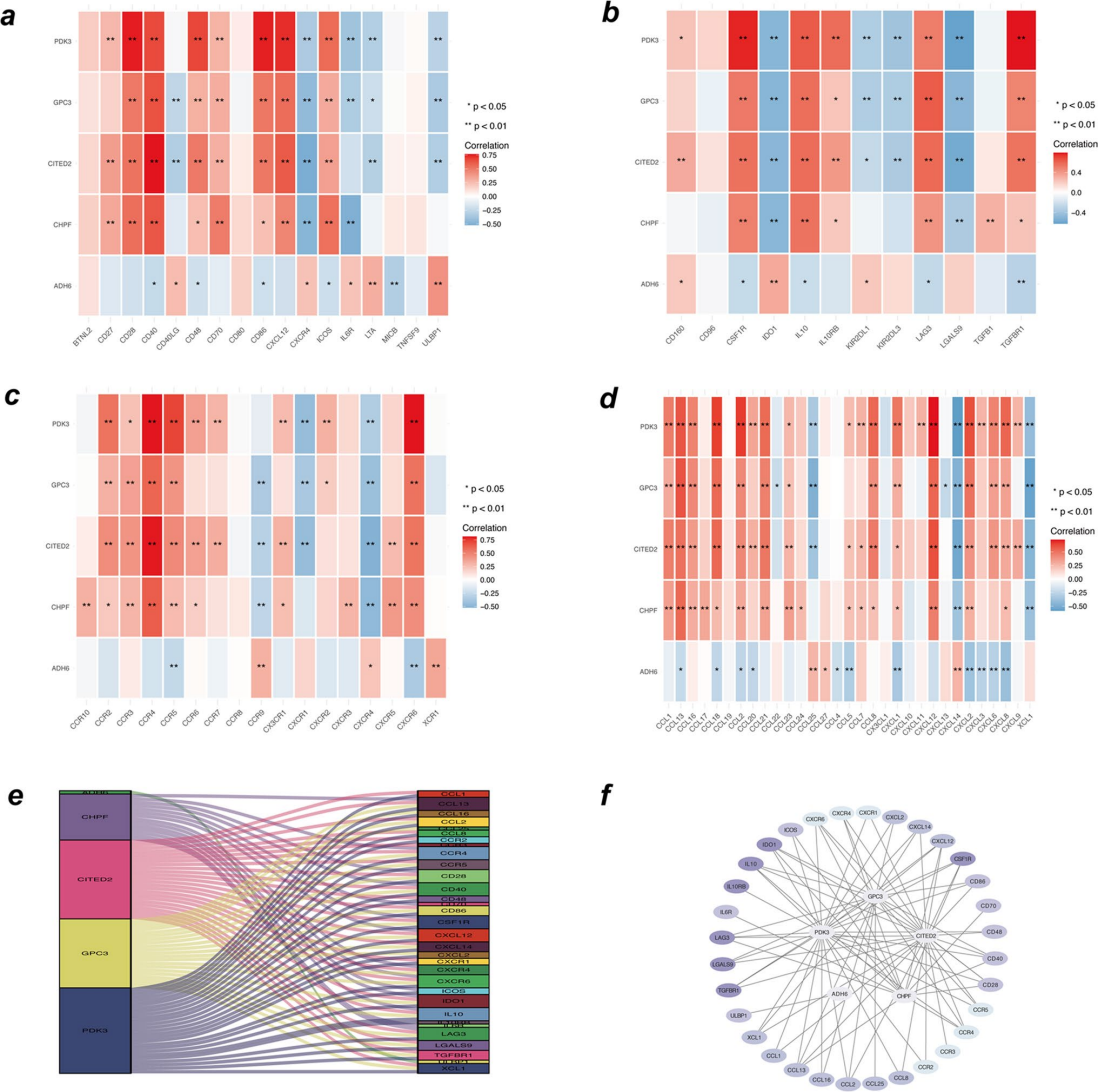
Association between the hub genes and immune cell infiltration. **a–d** Correlation between hub genes and chemokines, immune receptors, immunosuppressive factors, and immunostimulatory factors. **e** and **f** Protein–protein interaction plot of hub genes and immune-related molecules

The TISIDB database (an integrated repository portal for gene–immune system interactions) was then used to find associations between these five hub genes and various immunological variables, such as chemokines, receptors, immunosuppressive factors, and immunostimulatory factors. A correlation graph was created between immunological factors and key EMS genes (Fig. 6a–d). We selected immune factors associated with core genes (mean correlation coefficient > 0.4) and constructed an interaction network using Cytoscape and STRING (Fig. 6e, f). These findings indicated that key genes contribute significantly to the endometrial immune microenvironment.

### GSEA analysis of glycolysis-related hub genes

**Fig. 7:**
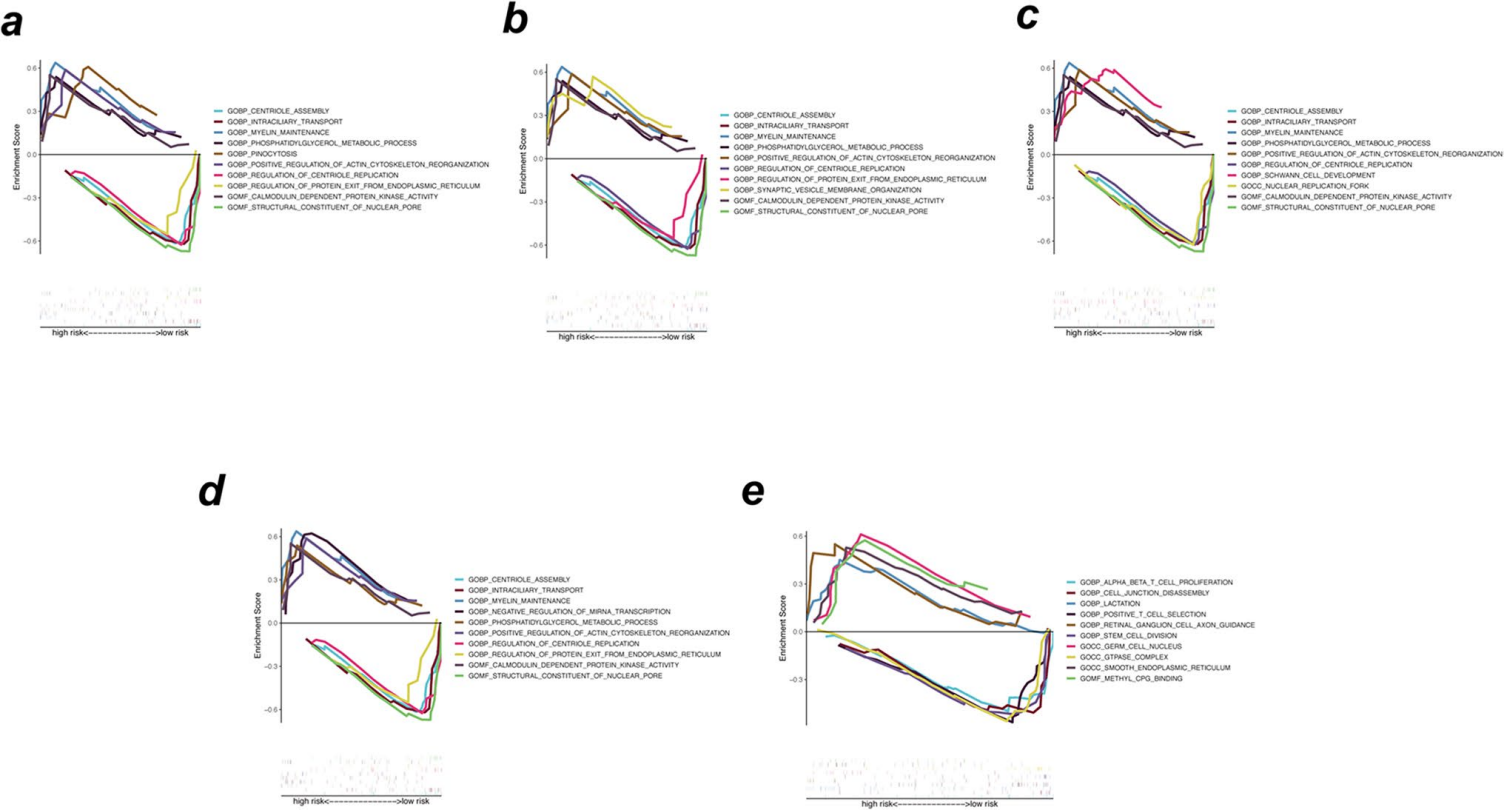
Enrichment analysis of pathway and Gene Ontology (GO) involved hub genes. **a–e** Gene Set Enrichment Analysis of CHPF, CITED2, GPC3, ADH6, and PDK3

**Fig. 8:**
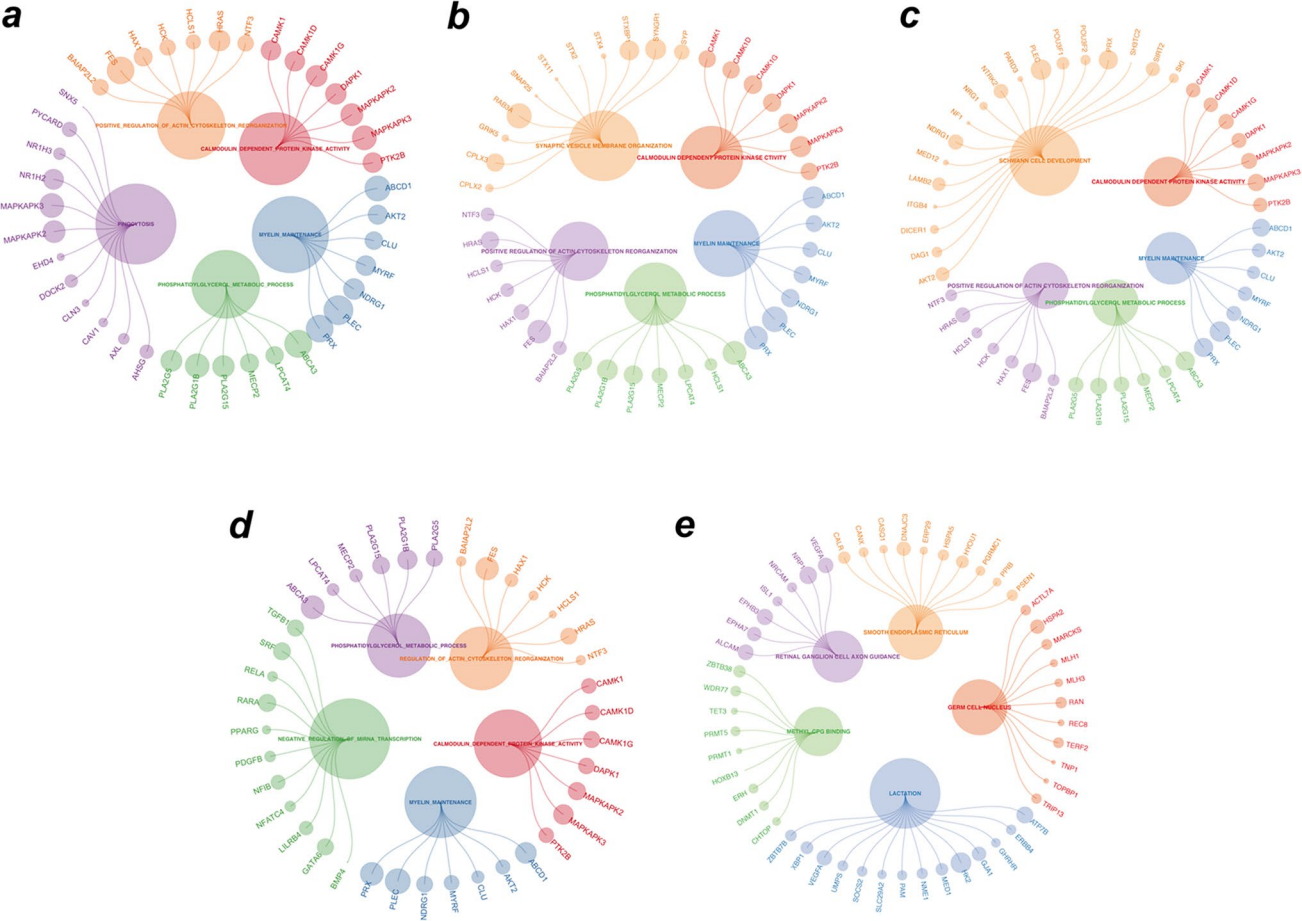
Molecular regulatory mechanism of core gene-related pathways and GO functional enrichment analyses. **a–e** GSEA-related ccgraph plot of CHPF, CITED2, GPC3, ADH6, and PDK3

We used GSEA on these five critical genes to investigate their activities and pathways. GSEA of five hub genes in the training set demonstrated that samples of these highly expressed hub genes (CHPF, CITED2, GPC3) were primarily enriched in “regulation of vesicle fusion,” “calmodulin-dependent protein kinase activity,” “myelin maintenance,” “phosphatidylglycerol metabolic process,” and “positive regulation of actin cytoskeleton recognition” related pathways. Additionally, samples with low expression of ADH6 were mainly enriched in “centriole assembly,” “structural constituent of nuclear pore,” “regulation of centriole replication,” “intraciliary transport,” and “regulation of protein exit from endoplasmic reticulum” (Fig. 7a–d). The results suggested that all the above genes were involved in biological functions, such as energy metabolism, material transport, and cell proliferation, which in turn contributed to the progression of EMS. Concurrently, the highly expressed PDK3 mainly participated in “methyl CPG binding,” “retinal ganglion cell axon guidance,” and “lactation”-related pathways (Fig. 7e). The molecular regulatory mechanisms of core genes are shown in a circle plot (Fig. 8a–e).

### GO similarity and coexpression of hub genes

**Fig. 9:**
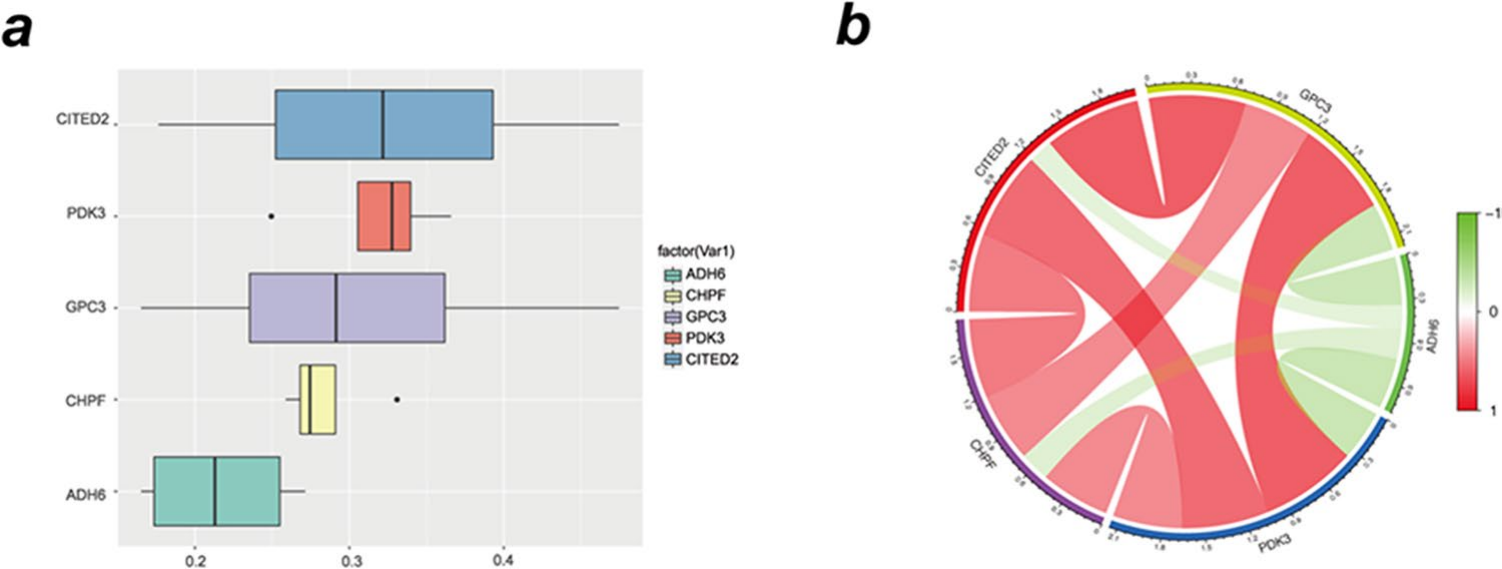
Closeness score of semantic similarities between GO terms and coexpression analysis of hub genes. **a** GO semantic similarity box plot of core genes. **b** The circos diagram depicts Pearson correlations between hub genes

To investigate the hub genes in EMS, we listed the key genes based on the average functional similarity links amongst the proteins. Amongst those genes, the scores of CITED2 (score: 0.324), PDK3 (score: 0.318), and GPC3 (score: 0.306) were the highest. The remaining two genes, CHPF (score: 0.285) and ADH6 (score: 0.216), were below 0.3 (Fig. 9a). Pearson analysis was performed to investigate correlations between hub genes. Compared to CHPF, CITED2, GPC3, and PDK3 were more strongly positively correlated with each other. However, only ADH6 remained negatively associated with other hub genes (Fig. 9b).

### RT-qPCR validation of the hub genes

**Fig. 10:**
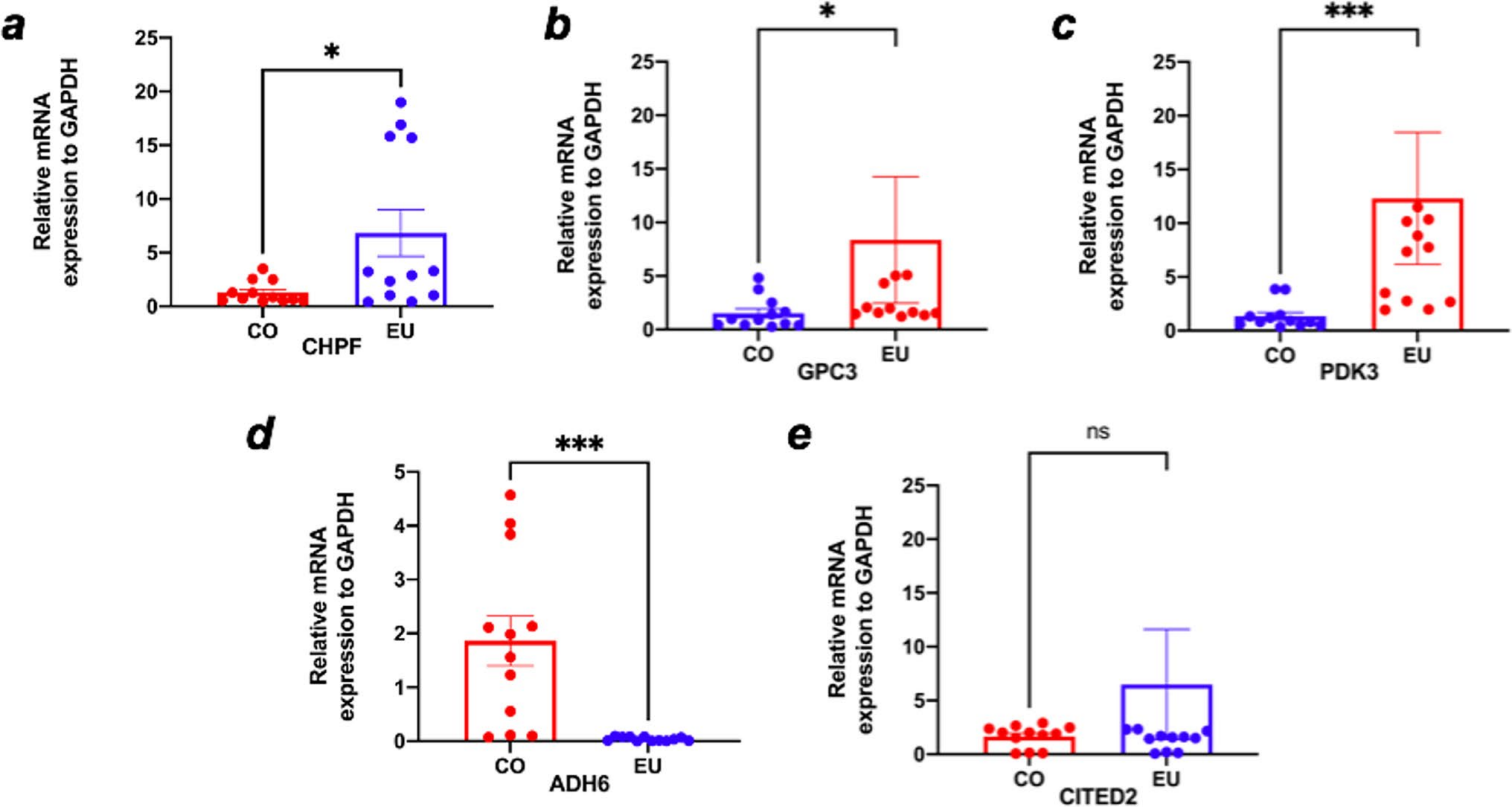
Expression of CHPF, GPC3, PDK3, ADH6, and CITED2 in eutopic endometrium samples collected from endometriosis patients and healthy controls as shown by RT-qPCR (*p < 0.05, **p < 0.01, ***p < 0.001)

We subsequently performed RT-qPCR experiments to explore the relative expression levels of the hub genes in the EMS and normal control groups. The research data demonstrated that the mRNA expression levels of GPC3, CHPF, and PDK3 in EMS were in contrast with those of the control (CHPF, *p* < 0.05; GPC3, *p* < 0.05; PDK3, *p* < 0.001). Conversely, the opposite effect was observed for ADH6 (*p* < 0.001). In addition, there was no significant difference in the levels of CITED2 between the EMS group and the control group (Fig. 10a–e). These five hub genes might function as potential diagnostic and prognostic biomarkers.

## Discussion

Endometriosis (EMS) is a systemic inflammatory disease caused by ectopic endometrium implantation and development outside the uterine cavity [2]. Recent studies have focused on glycolytic pathways in cancer cell growth and invasion. Endometrium cells, like cancer cells, have the ability to switch energy metabolism from mitochondrial oxidative phosphorylation (OXPHOS) to aerobic glycolysis to reduce ROS generation and enhance survival [4]. It has been reported that glucose metabolism and energy production in ectopic endometriotic cells under hypoxia influence the incidence and invasion of endometriosis [24, 25]. Nevertheless, studies involving the Warburg effect in eutopic endometrial cells are still lacking. Increasing findings suggest that changes in glycolytic metabolism in the endometrial microenvironment may have impacts on immune cell infiltration and other anti-immune processes, but the specific mechanism remains to be explored.

Early prediction and detection of EMS can lead to early interventions and improve treatment outcomes. Therefore, the identification of possible biomarkers for predicting EMS is crucial. In recent years, advances in machine learning techniques and the availability of gene expression data in public databases have provided a new approach to identifying biomarkers for disease detection.

In this work, we identified five glycolysis-related potential genes (CHPF, CITED2, PDK3, GPC3, and ADH6) through LASSO regression analysis and the RF method. The CIBERSORT algorithm was then used to perform a deconvolution study of the immune microenvironment to determine the fraction of immune cells in EMS. The relationship between core genes and other immunomodulators, as well as the majority of chemokines and receptors mentioned in TISIDB, was then investigated. The profiles of the five hub genes were identified using GO semantic similarity and GSEA. The risk score based on the five glycolysis-related markers was then used to create a nomogram, and the nomogram had good predictive performance.

Chondroitin polymerizing factor (CHPF) is a type II transmembrane protein that is essential for chondroitin sulfate (CS) production. Many cell biological functions, such as cell adhesion, cell differentiation, and neural network creation, rely on CS [26]. Li et al. [27] reported that the expression of CHPF was linked to immune cells and various immune factors. At present, most studies focus on the function of CHPF in cancers, and little has been studied in endometriosis.

CBP/p300-interacting-transactivator-with-an-ED-rich-tail 2 (CITED2) is a transcriptional regulator that regulates biological functions by coactivating or repressing multiple transcription factors [28]. The glycolytic gene CITED2 is also a hypoxia-related gene. It has been reported that CITED2 is associated with primary ovarian insufficiency [29].

Glypican-3 (GPC3) is a membrane-associated proteoglycan involved in cell growth, differentiation, and migration. The specific expression of GPC3 in tumour cells has received much attention [30]. In a Canadian patient cohort, high membranous GPC3 expression was found in 20% of endometriosis-associated ovarian clear cell carcinomas (OCCCs) [31].

Pyruvate dehydrogenase kinase 3 (PDK3) is a member of the PDK family, which contains PDK1, PDK2, PDK2, and PDK4. PDK3 mainly contributes to metabolic switching and cell survival under hypoxia, similar to CITED2 [32]. Simultaneously, PDK3 plays a crucial role in cancers and has been regarded as a promising target for cancers [33]. Our results found that PDK3 was overexpressed in the eutopic endometrium in women with endometriosis. However, the function of PDK3 in endometriosis is unclear.

The serum levels of alcohol dehydrogenase 6 (ADH6) have been shown in numerous studies to be a potential diagnostic marker in cancers. It has been substantiated that ADH6 is involved in the P450-related pathway and biological processes linked to the progression and treatment of pancreatic cancer [34]. Its involvement in endometriosis biology, however, is unknown.

The biological behaviour of endometriosis is similar to that of malignant tumours. Glucose is the most readily available nutrient for cancer cells, but it is also required for T cell activation, differentiation, and function. Proliferating tumour cells that consume a large amount of extracellular glucose secrete lactic acid into the cancer microenvironment. Lactate was later discovered to inhibit monocyte migration and cytokine release as well as promote resident macrophage polarization to the tumour-associated macrophage 2 (TAM2) phenotype, resulting in tumour progression and immune escape [35].

Although endometriosis is a benign illness, it exhibits neoplastic traits such as inflammation and tissue invasion [36]. Therefore, we speculate that the same biological process between glycolysis and the immune environment occurs in endometriosis [35]. The abundances of follicular helper T cells, T cell regulators (Tregs), M0 macrophages, activated NK cells, monocytes, activated dendritic cells, and resting mast cells (MCs) were higher in the eutopic endometria of women with endometriosis than in those of normal controls in our study. Tfh cells are a kind of CD4^+^ T cell that plays a critical role in the adaptive immune response. The roles of Tfh cells in endometriosis have received little attention [37]. T cell regulators (Tregs) are increased in the endometrium of women with and without disease, according to most research. However, there is still debate [38]. Previous research has shown that in the proliferative phase of endometriosis, more macrophages (Møs) and activated dendritic cells are found in the endometrium of women with endometriosis, regardless of the hormonal milieu [38]. Currently, it appears that uterine natural killer (uNK) cells from women with endometriosis are immature and that uNK cytotoxic activity could be an indicator of endometriosis-related infertility and recurrent miscarriage, although the absolute numbers are the same as in normal endometrium [39]. Others have found higher mast cell infiltration in the endometrium in women with illness, as well as enhanced MC activation in ectopic lesions, but activated MCs in eutopic endometrium were rarely found [40]. We also found increased monocytes in the eutopic endometrium of women with endometriosis, and we infer that monocytes are largely recruited as a source of monocyte-derived macrophages [41].

Next, we analysed the correlation between hub glycolysis-related gene expression and infiltration of various immune cell types. Likewise, we investigated the relationship between five hub genes, various immunomodulators, chemokines, and receptors listed in TISIDB. Finally, according to GSEA, hub genes are primarily involved in biological processes, such as energy metabolism, material transport, and cell proliferation, which in turn contribute to the progression of EMS. Moreover, compared to CHPF, CITED2, GPC3, and PDK3 were more strongly positively correlated with each other. ADH6 remained negatively associated with other hub genes.

The RT-qPCR results were consistent with bioinformation findings. ADH6 levels were significantly lower in the EMS group than in the control group, whilst mRNA expression levels for GPC3, PDK3, and CHPF were significantly higher in the EMS patients than in the control group.

We discovered five glycolysis-related hub genes that are closely associated with the molecular mechanism of EMS using bioinformatic analysis, verified the biological functions and important pathways of the hub genes, and performed immune cell infiltration and correlation analysis for the target core genes. However, this work has certain limitations. It is just a proof of concept, and more in vitro and in vivo experiments are needed to confirm our findings and investigate the mechanisms by which glycolysis-related genes regulate the infiltration of immune cells.

## Conclusions

In conclusion, we discovered five glycolysis-related genes in endometriosis and developed a model for EMS assessment. Based on numerous bioinformatics techniques, we discovered hub genes in EMS and their correlation with infiltrating immune cells, as well as correlations between 22 immune cell subpopulations. Meanwhile, GSEA and GO similarity analysis revealed more specific mechanisms. Selected genes could be candidate predictive markers and potential therapeutic targets for EMS, but the exact mechanisms of glycolysis-related genes and the immune environment (including immune cells and immune factors) in EMS should be further explored.

## Supplementary Information

Below is the link to the electronic supplementary material.

Supplementary file1 (DOCX 4563 KB)

Supplementary file2 (DOCX 4524 KB)

Supplementary file3 (XLS 0 KB)

Supplementary file4 (XLS 0 KB)

Supplementary file5 (XLS 29 KB)

## Data Availability

The datasets generated during and/or analysed during the current study are available in the GEO. The data supporting this study’s findings are available from the corresponding author upon reasonable request. The names of the repository/repositories and accession number(s) are listed below:https://www.ncbi.nlm.nih.gov/geo/query/acc.cgi?acc=GSE25628https://www.ncbi.nlm.nih.gov/geo/query/acc.cgi?acc=GSE51981https://www.ncbi.nlm.nih.gov/geo/query/acc.cgi?acc=GSE7846https://www.ncbi.nlm.nih.gov/geo/query/acc.cgi?acc=GSE7305https://www.ncbi.nlm.nih.gov/geo/query/acc.cgi?acc=GSE120103https://www.ncbi.nlm.nih.gov/geo/query/acc.cgi?acc=GSE6364

## Abbreviations

EMS: Endometriosis
NCBI: National Center For Biotechnology Information
GEO: Gene Expression Omnibus
GSEA: Gene Set Enrichment Analysis
MSigDB: Molecular Signatures Database
LASSO: Least absolute shrinkage and selection operator
RF: Random forest
TME: Tumour microenvironment
DCA: Decision curve analysis
ROC: Receiver operating characteristic curve
AUC: Area under curve
CHPF: Chondroitin polymerizing factor
CITED2: CBP/p300-interacting-transactivator-with-an ED-rich-tail 2
GPC3: Glypican-3
PDK3: Pyruvate dehydrogenase kinase 3
ADH6: Alcohol dehydrogenase 6
Tregs: T cell regulators
Møs: Macrophages
uNK: Uterine natural killer
