# Supplementary material for: Establishment of a novel glycolysis-immune-related diagnosis gene signature for endometriosis by machine learning
Source: J Assist Reprod Genet. 2023 Mar 17;40(5):1147–61. doi: 10.1007/s10815-023-02769-0 (PMC10239430; doi:10.1007/s10815-023-02769-0)
Supplement: Supplementary file 1 — Supplementary file1 (DOCX 4563 KB) [file 10815_2023_2769_MOESM1_ESM.docx]

**Figure SI**

**
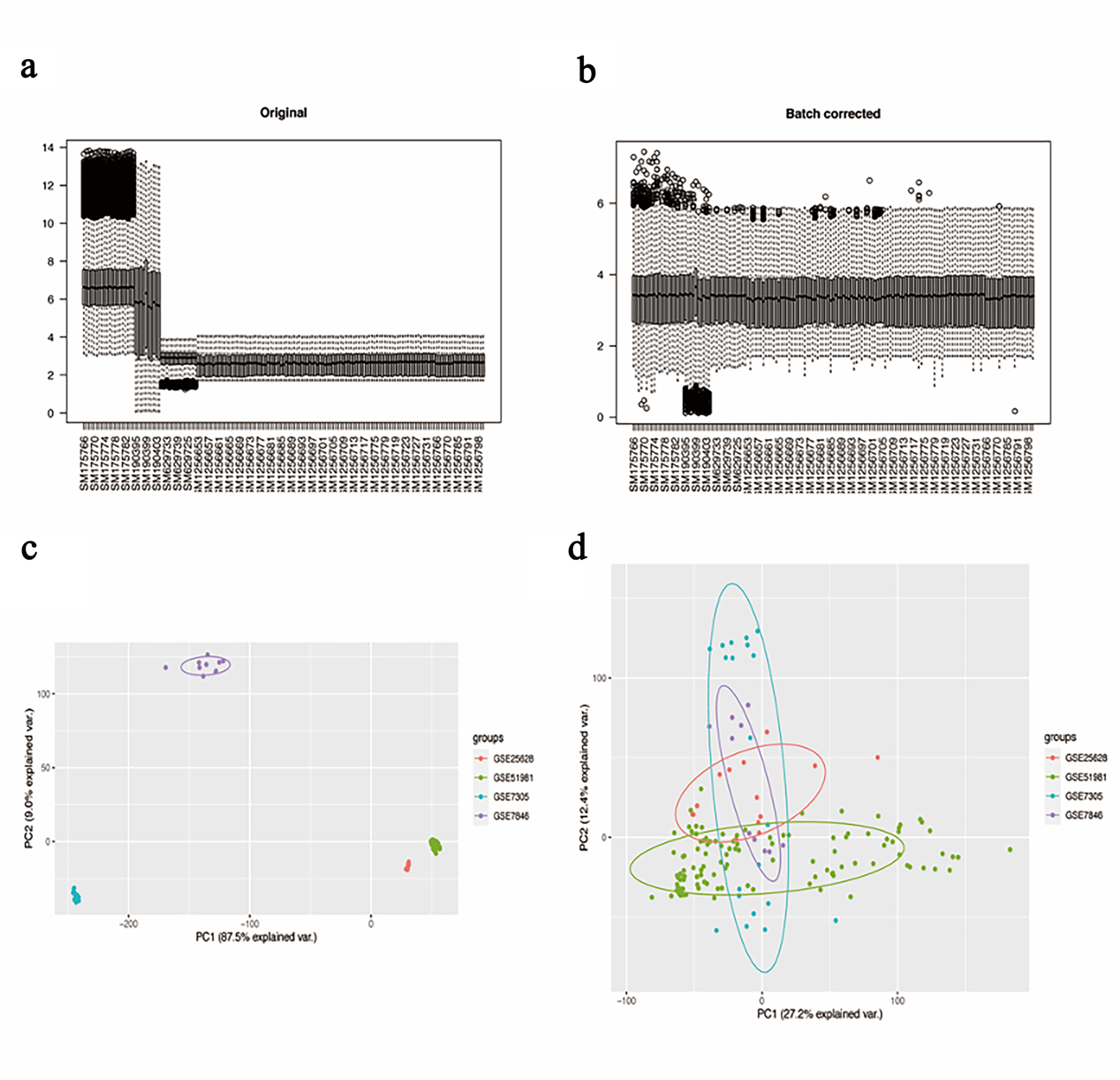
**

**Fig SI** Batch effect removal and normalization of the training set (a-b) Box plot of data in the training set before and after normalized (c-d) PCA results before and after batch effect removal
