# Supplementary material for: Establishment of a novel glycolysis-immune-related diagnosis gene signature for endometriosis by machine learning
Source: J Assist Reprod Genet. 2023 Mar 17;40(5):1147–61. doi: 10.1007/s10815-023-02769-0 (PMC10239430; doi:10.1007/s10815-023-02769-0)
Supplement: Supplementary file 2 — Supplementary file2 (DOCX 4524 KB) [file 10815_2023_2769_MOESM2_ESM.docx]

**Figure SII**

**
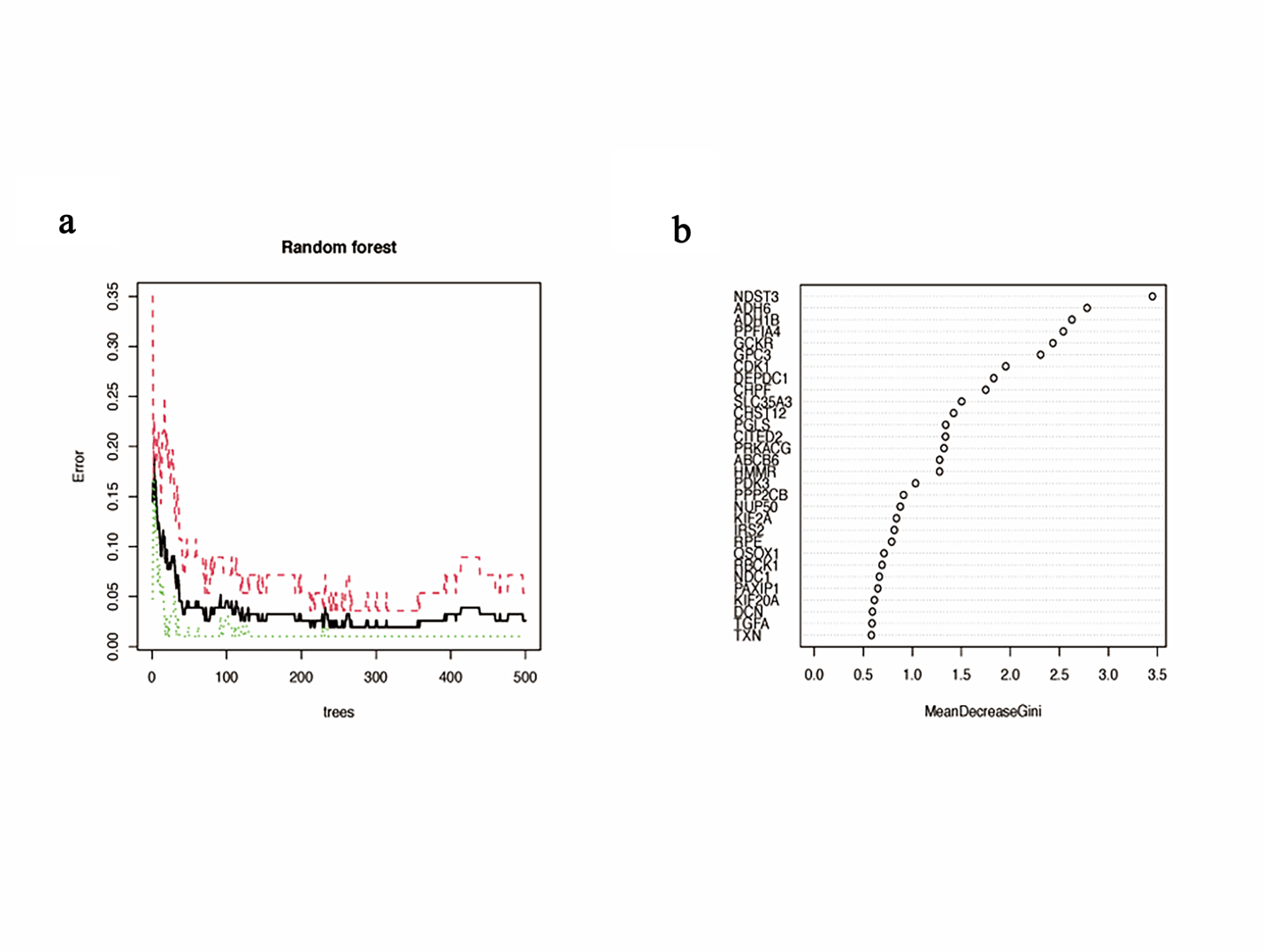
**

**Fig SII** Results of the Random Forest (a) Error rate of the Random Forest (b) the rank of gene importance in the Random Forest
